# Volumetric Brain Loss Correlates With a Relapsing MOGAD Disease Course

**DOI:** 10.3389/fneur.2022.867190

**Published:** 2022-03-24

**Authors:** Ariel Rechtman, Livnat Brill, Omri Zveik, Benjamin Uliel, Nitzan Haham, Atira S. Bick, Netta Levin, Adi Vaknin-Dembinsky

**Affiliations:** ^1^Department of Neurology and Laboratory of Neuroimmunology and the Agnes-Ginges Center for Neurogenetics, Hadassah-Medical Center, Ein–Kerem, Faculty of Medicine, Hebrew University of Jerusalem, Jerusalem, Israel; ^2^Functional Imaging Unit, Department of Neurology, Hadassah-Hebrew University Medical Center, Jerusalem, Israel

**Keywords:** MOGAD, brain MRI, relapsing MOGAD, brain volume, brain atrophy

## Abstract

**Background:**

Myelin oligodendrocyte glycoprotein antibody disorders (MOGAD) have evolved as a distinct group of inflammatory, demyelinating diseases of the CNS. MOGAD can present with a monophasic or relapsing disease course with distinct clinical manifestations.However, data on the disease course and disability outcomes of these patients are scarce. We aim to compare brain volumetric changes for MOGAD patients with different disease phenotypes and HCs.

**Methods:**

Brain magnetic resonance imaging (MRI) scans and clinical data were obtained for 22 MOGAD patients and 22 HCs. Volumetric brain information was determined using volBrain and MDbrain platforms.

**Results:**

We found decreased brain volume in MOGAD patients compared to HCs, as identified in volume of total brain, gray matter, white matter and deep gray matter (DGM) structures. In addition, we found significantly different volumetric changes between patients with relapsing and monophasic disease course, with significantly decreased volume of total brain and DGM, cerebellum and hippocampus in relapsing patients during the first year of diagnosis. A significant negative correlation was found between EDSS and volume of thalamus.

**Conclusions:**

Brain MRI analyses revealed volumetric differences between MOGAD patients and HCs, and between patients with different disease phenotypes. Decreased gray matter volume during the first year of diagnosis, especially in the cerebrum and hippocampus of MOGAD patients was associated with relapsing disease course.

## Introduction

Myelin oligodendrocyte glycoprotein (MOG) antibody disorders (MOGAD) are a newly recognized group of inflammatory demyelinating diseases of the central nervous system (CNS), characterized by the presence of immunoglobulin G (IgG) antibodies against MOG presented on myelin sheaths (1, 2). MOGAD are predominantly associated with acute disseminated encephalomyelitis (ADEM) in young children and with optic neuritis (ON) and myelitis in adults, and have lower prevalence in cases of encephalitis and seizures. This has evolved into a new inflammatory CNS disease entity that is distinct from both multiple sclerosis (MS) and neuromyelitis optica spectrum disorders (NMOSD), and is characterized by younger age at onset, equal frequency in males and females, and an optic nerve preference (3, 4).

MOGAD occurs in all decades of life and can present with a monophasic or relapsing course. A relapse pattern has been reported in 44–83% of adults with MOGAD (5). Young adults mostly present with relapsing disease course, while early and late-onset MOGAD mainly presents with a monophasic disease course (6). Children that experience a relapsing course (20-34%) usually present as ADEM, followed by one or multiple episodes of ON, multiphasic disseminated encephalomyelitis, or relapsing NMOSD-like syndromes (7). The current evidence is controversial regarding whether higher MOG antibody levels predict a relapsing course (6). Predicting the disease course will dictate the patient's management (8).

MOGAD lesions are characterized by demyelination, MOG loss, and relative preservation of axons and oligodendrocytes. The cellular infiltrates consist of macrophages/microglia, T-cells (CD4 dominance), and granulocytes. Humoral immunity, evidenced by B cells, IgG and perivascular deposits of activated complement, was observed, although in lower levels than in AQP4 antibody-positive NMOSD (9). Cortical demyelination occurs relatively frequently in MOGAD patients with brain involvement and is often topographically associated with meningeal infiltration (10).

Magnetic resonance imaging (MRI) is a critically important tool for diagnosis and differentiation of demyelinating disorders. Prognosis, disease monitoring and treatment changes are based on clinical symptoms and neuroimaging findings (11). MRI allows whole-brain volume to be measured, as well as the volume of brain lobes and gyri. In MS, whole brain atrophy is considered a good predictor of long-term clinical disability (12). Two-thirds of MOGAD cases have normal brain MRI, however, when lesions exist, they tend to be with a predilection for the brainstem and infratentorial regions. The lesions are mostly bilateral, affecting the deep white matter (13). MRI findings in MOG encephalomyelitis/encephalitis are usually described as an ADEM-like pattern with diffuse signal changes noted in the cortical gray matter/subcortical white matter, and deep white and gray matter (4). In the majority of cases, following clinical recovery there is complete resolution of all MRI abnormalities (14). Extensive lesions can be seen in the optic nerve, predominantly involving the anterior segments of the optic nerve, sparing chiasm and optic tracts (14).

So far, few studies have examined brain volumes of patients with MOGAD. In this study, we analyzed high-resolution MRI data of MOGAD patients with different disease phenotypes and HCs using volBrain and MDbrain analysis software.

## Methods

### Approvals

The study was approved by Hadassah Medical Organization's Ethics Committee (reference no. HMO-20-0644). Given the study design, the Hadassah Medical Organization's Ethics Committee has determined that written consent wasn't required. We confirm that all experiments were performed in accordance with relevant guidelines and regulations.

### MOG Antibody Testing

Serum samples were tested for MOG-IgG using the Euroimmun commercial biochip immunofluorescence cell-based assay [IIFT: Myelin-oligodendrocyte glycoprotein (MOG) product numb 1156]. Briefly, specific antibodies from the diluted patient sample bind to the solid-phase MOG-bound antigens. In the next step, a fluorescein (FITC)-labeled antibody (conjugate) binds to the specific antibodies from the patient sample. By excitation with the respective wavelength, the complex can be made visible at the fluorescence microscope (15).

### MRI Data Acquisition, Processing, and Analysis

Three-dimensional T1-weighted images were acquired mainly using 3 Tesla MRI scanner. Eight of the patients were obtained with 1.5 Tesla. All the MRIs of MOGAD patients were acquired using Demyelination protocol (16). Volumetric data were extracted using the volBrain (http://volbrain.upv.es) and MDbrain (https://grand-challenge.org/aiforradiology/product/mediaire-mdbrain/) platforms. volBrain software contains advanced pipelines and automatically provides volumetric information of the brain MR images at different scales (17). Validation analysis was performed using MDbrain software, an artificial intelligence-based software tool for volumetric brain analysis and characterization of white matter lesions. It quantifies volumetric values and codes deviations from a normal database. MDbrain analysis calculates the coded deviations from normal database, and is available for total brain, white and gray matter, cerebrum, hippocampus and cerebellum.

Cerebellar gray matter was determined using the multi-atlas segmentation tool CEREbellum Segmentation (CERES) (18). HIPS is a pipeline of volBrain for segmenting the hippocampus and its subfields (19).

No differences were found when comparing brain volume levels between the two MRI scanners (Supplementary Table S1).

### Statistical Analysis

Distribution was tested using the Kolmogorov–Smirnov test. Due to the normal distribution, we used Student's *t*-test to assess differences between two independent variables.

Spearman correlation study was used to assess the correlation between EDSS levels, and number of relapses to brain volume. Differences were considered significant at *P* ≤ 0.05.

## Results

### Patients

The patient cohort included 22 patients [18 females, 4 males; average age: 33.10 ± 17.19 years; mean disease duration 3.51 ± 3.24 years; mean Expanded Disability Status Scale (EDSS), 1.10 ± 1.30; average relapse number: 1.62 ± 1.07; mean duration between first episode and MRI scan: 2.01 ± 2.89 years]. All patients are MOG-IgG seropositive (Demographic and clinical data are presented at Table 1A). A group of 22 healthy individuals served as controls (average age: 38.23 ± 12.58; Female:Male ratio: 17:5).

**Table 1A:** Clinical and imaging data of MOGAD patients.

**#**	**Myelitis**	**ON**	**EDSS**	**Number of relapses**	**Number of relapses before MRI**	**Brain MRI**	**Disease duration (months)**	**Duration from first episode to MRI (months)**	**Treatment**	**OCB**
1	No	BIL ON	1	2	2	Encephalitis-like^a^	48	30	B-cell depletion	Negative
2	No	Uni ON	1	2	1	Normal	30	11	Prednisone	Positive
3	Yes	Uni ON	0	3	2	NMO-like^b^	42	11	Methotrexate	Negative
4	Yes	No	0	1	1	Normal	36	3	No Treatment	Negative
5	No	BIL ON	2	4	2	MS-like^c^	24	12	B-cell depletion	Positive
6	No	No	0	1	1	Non-specific lesions^d^	18	18	No Treatment	Positive
7	Yes	No	5	1	1	Normal	72	58	No Treatment	Negative
8	Yes	Uni ON	4	2	2	Normal	18	11	No Treatment	Negative
9	No	Uni ON	1	1	1	Normal	42	9	No Treatment	Negative
10	Yes	No	1	1	1	Normal	54	33	No Treatment	Negative
11	No	BIL ON	1	2	1	Non-specific lesions^e^	30	6	Prednisone	Negative
12	No	BIL ON	1	1	1	NMO-like^f^	132	84	No Treatment	NA
13	No	BIL ON	0	1	1	Normal	30	At first episode	No Treatment	Negative
14	No	Uni ON	1	2	2	Normal	120	120	B-cell depletion Prednisone	NA
15	Yes	No	0	1	1	Area postrema lesion	42	11	No Treatment	NA
16	No	Uni ON	0	4	4	Normal	132	96	Cellcept	NA
17	No	BIL ON	1	1	1	Normal	8	At first episode	No Treatment	Negative
18	No	Uni ON	1	1	1	Normal	5	At first episode	No Treatment	Negative
19	No	Uni ON	2	1	1	Normal	5	At first episode	No Treatment	Negative
20	No	Uni ON	0	1	1	Normal	4	At first episode	No Treatment	Negative
21	Yes	No	2	1	1	Normal	3	At first episode	No Treatment	Positive
22	No	Uni ON	0	1	1	Normal	108	At first episode	No Treatment	Negative

*Uni ON, unilateral optic neuritis; BIL ON, bilateral optic neuritis; NMO, neuromyelitis optica; OCB, oligoclonal bands; NA, data not aviable; EDSS, estimated disability status scale*.

*^a^Prolongation of MRI signal at frontal, parietal and temporal cortex. In addition, prolongation of MRI signal in pons and brain stem*.

*^b^Abnormal signal intensity involving the medulla and pons*.

*^c^Multiple white matter lesion within the white matter of the cerebral hemispheres. One lession located in the periventricular white matter of the occipital lobe*.

*^d^Multiple bilateral hyperintense non-enhancing T2/Flair Foci involving the supratentorial periventricular and centrum locations.*

*^e^Non-specific lesions at subcortical frontal white matter.*

*^f^Prolongation of T2/Flair in subcortical supratentorial deep white matter*.

The patient cohort composed of 8 monophasic patients, 8 relapsing patients and 6 patients classified as unknown (disease duration <2.5 years with one relapse). Patients were classified as having a monophasic course after at least 2.5 years of follow-up without relapse (20).

There were no significant differences between the monophasic and relapsing group in regards to age (36.50 ± 15.46 vs. 32.88 ± 20.29, *p* = 0.69), gender (Female:Male ratio: 6:2 vs. 7:1, *p* = 0.52), disease duration at MRI scan (2.16 ± 2.61 vs. 3.19 ± 3.70, *p* = 0.53), EDSS (1.13 ± 1.64 vs. 1.25 ± 1.28, *p* = 0.87), and scans performed in 1.5 vs. 3 Tesla (4:4 vs. 3:5, *p* = 0.61) (Demographic and clinical data of the patients are presented at Table 1B).

**Table 1B:** Clinical and demographic data of monophasic and relapsing MOGAD patients.

	**Monophasic**	**Relapsing**	***P*-value**
Age (years)	36.50 ± 15.46	32.88 ± 20.29	0.69
Gender (female:male ratio)	6:2	7:1	0.52
Disease duration at MRI scan (years)	2.16 ± 2.61	3.19 ± 3.70	0.53
EDSS	1.13 ± 1.64	1.25 ± 1.28	0.87
Relapse number	1	2.75 ± 1.04	<0.001

*EDSS, estimated disability status scale*.

### Brain Volume Loss in MOGAD Patients During the First Year After Diagnosis

**Figure 1:**
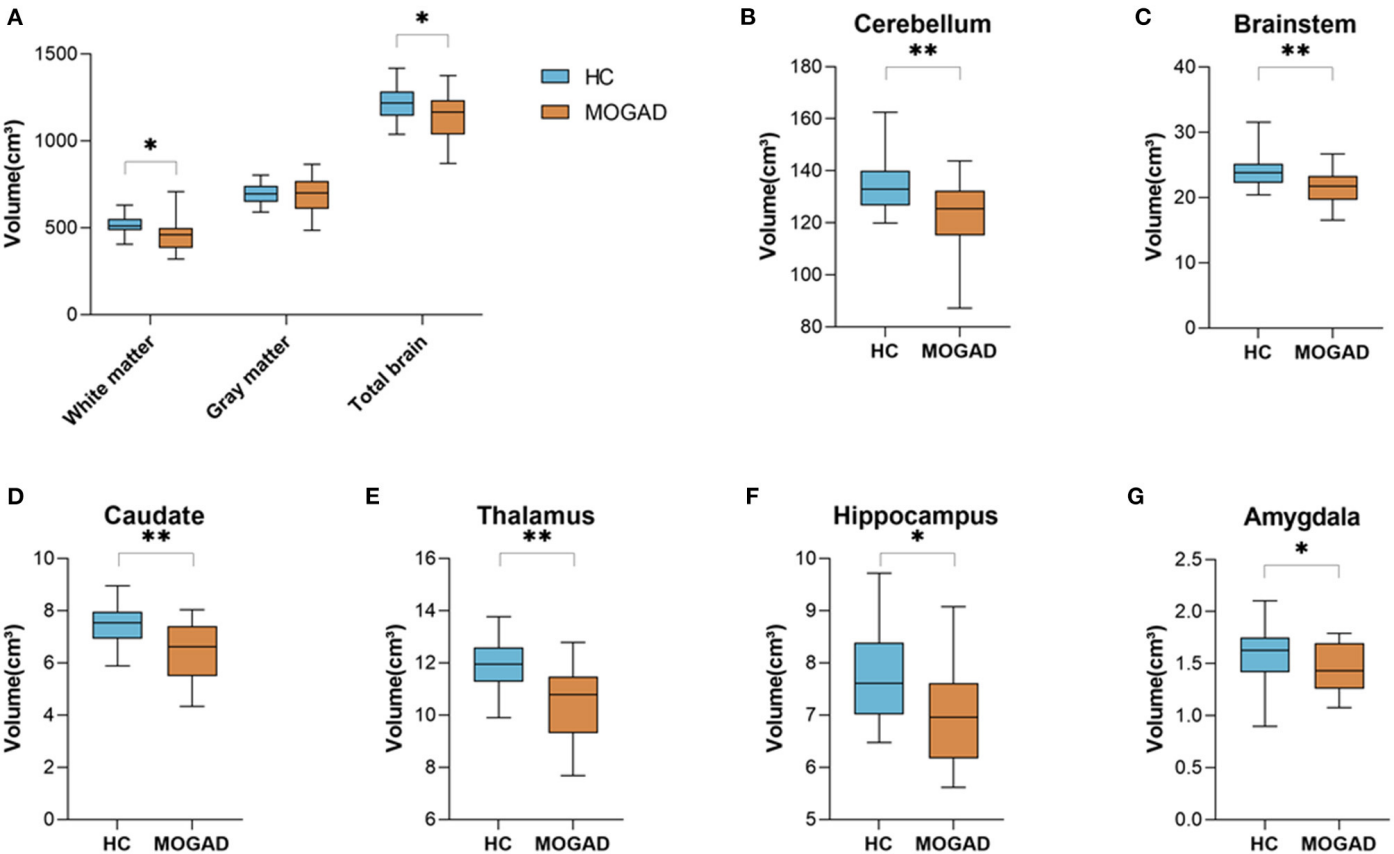
Decreased brain volume in MOGAD patients compare to HCs. Brain volume of HCs (*n* = 22) and MOGAD patients (*n* = 22) analyzed by Volbrain software. **(A)** Volume of white matter (512.56 ± 57.33 vs. 458.68 ± 89.60, *p* = 0.018), gray matter (699.97 ± 59.45 vs. 681.66 ± 106.70, *p* = 0.476) and total brain (1,214.14 ± 105.65 vs. 1,139.91 ± 135.15, *p* = 0.048), and volume of **(B)** cerebellum (134.64 ± 10.28 vs. 124.17 ± 12.42, *p* = 0.007), **(C)** brainstem (23.96 ± 2.55 vs. 21.61 ± 2.45, *p* = 0.003), **(D)** caudate (7.46 ± 0.79 vs. 6.56 ± 1.03, *p* = 0.001), **(E)** thalamus (11.97 ± 0.98 vs. 10.74 ± 1.72, *p* = 0.008), **(F)** hippocampus (7.78 ± 0.83 vs. 6.86 ± 1.27, *p* = 0.011), and **(G)** amygdala (1.60 ± 0.27 vs. 1.37 ± 0.36, *p* = 0.024) of MOGAD patients and HCs. MOGAD, Myelin oligodendrocyte glycoprotein antibody disorders; HCs, healthy controls. *p* * ≤ 0.05, ** ≤ 0.01.

We analyzed brain MRI scans of 22 MOGAD patients and 22 HCs and found a significant decreased total brain volume in MOGAD patients compared to HCs (1,214.14 ± 105.65 vs. 1,139.91 ± 135.15, *p* = 0.048). Specifically, there was a significant decrease in white matter volume in MOGAD patients (512.56 ± 57.33 vs. 458.68 ± 89.60, *p* = 0.018) (Figure 1A). Looking at the deep gray matter structures of MOGAD patients we observed significant decrease volume in the cerebellum (134.64 ± 10.28 vs. 124.17 ± 12.42, *p* = 0.007), brainstem (23.96 ± 2.55 vs. 21.61 ± 2.45, *p* = 0.003), caudate (7.46 ± 0.79 vs. 6.56 ± 1.03, *p* = 0.001), thalamus (11.97 ± 0.98 vs. 10.74 ± 1.72, *p* = 0.008), hippocampus (7.78 ± 0.83 vs. 6.86 ± 1.27, *p* = 0.011), and amygdala (1.60 ± 0.27 vs. 1.37 ± 0.36, *p* = 0.024) compared to HCs (Figures 1B–G).

**Figure 2:**
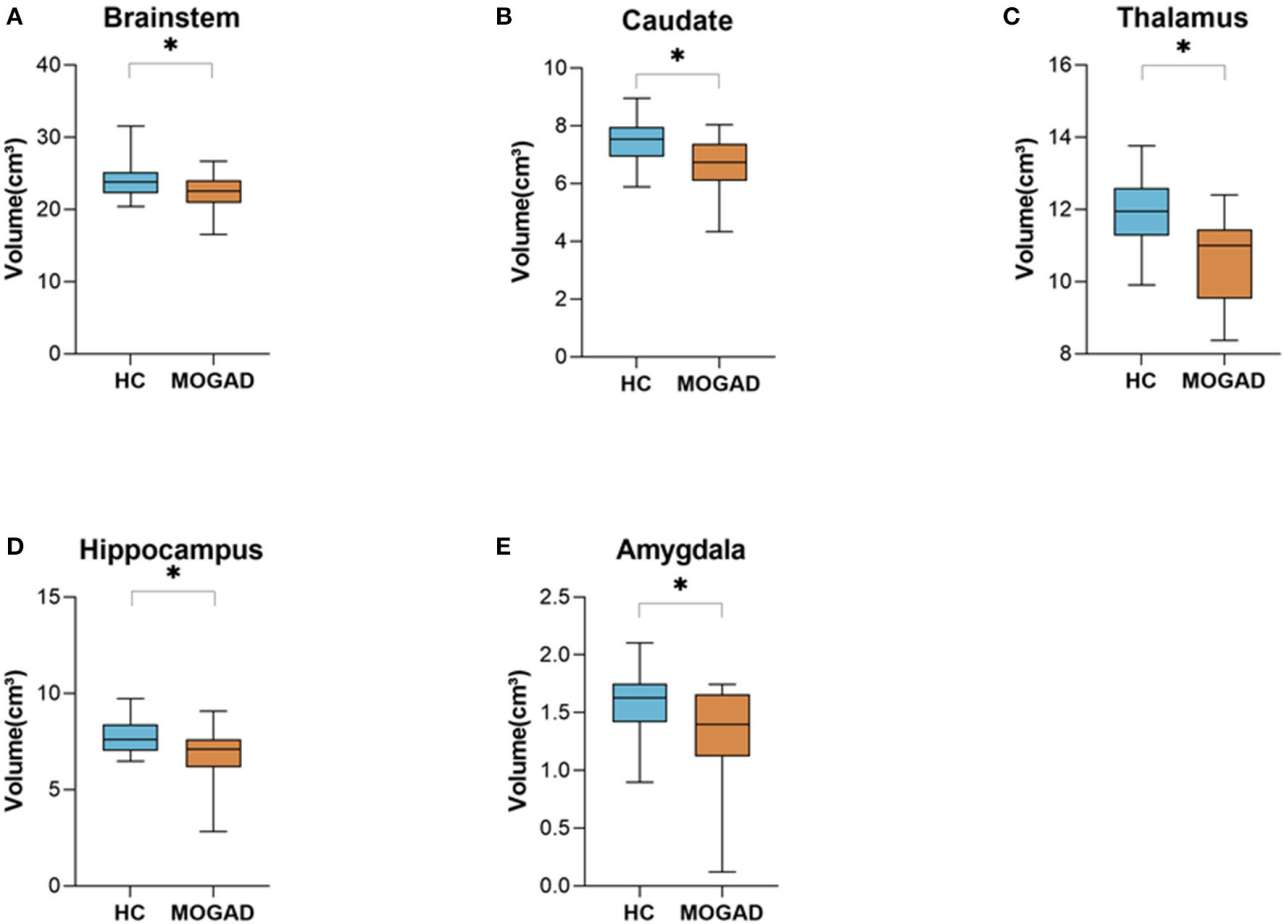
Decrease brain volume of MOGAD patients during the first year of diagnosis. Brain volume of HCs (*n* = 22) and MOGAD patients during the first year of diagnosis (*n* = 15) analyzed by Volbrain software. **(A)** Volume of brainstem (23.96 ± 2.49 vs. 22.13 ± 2.48, *p* = 0.035), **(B)** caudate (7.46 ± 0.79 vs. 6.68 ± 1.03, *p* = 0.010), **(C)** thalamus (11.92 ± 0.99 vs. 10.99 ± 1.67, *p* = 0.040), **(D)** hippocampus 7.78 ± 0.83 vs. 6.82 ± 1.41, *p* = 0.021), and **(E)** amygdala (1.60 ± 0.27 vs. 1.32 ± 0.41, *p* = 0.021) of MOGAD patients during the first year of diagnosis and HCs. MOGAD, Myelin oligodendrocyte glycoprotein antibody disorders; HCs, healthy controls. *p* * ≤ 0.05.

Limiting the analyses to 15 brain MRI scans of MOGAD patients performed during the first year after diagnosis yielded similar results. Significantly decreased volume of deep gray matter structures compare to HCs: brainstem (23.96 ± 2.49 vs. 22.13 ± 2.48, *p* = 0.035), caudate (7.46 ± 0.79 vs. 6.68 ± 1.03, *p* = 0.010), thalamus (11.92 ± 0.99 vs. 10.99 ± 1.67, *p* = 0.040), hippocampus (7.78 ± 0.83 vs. 6.82 ± 1.41, *p* = 0.021), and amygdala (1.60 ± 0.27 vs. 1.32 ± 0.41, *p* = 0.021) (Figures 2A–E) (Volumetric MRI parameters are presented in Supplementary Table S2,3) .

### Volumetric Brain Loss Evident at Diagnosis Correlates With Relapsing MOGAD Disease Course

**Figure 3:**
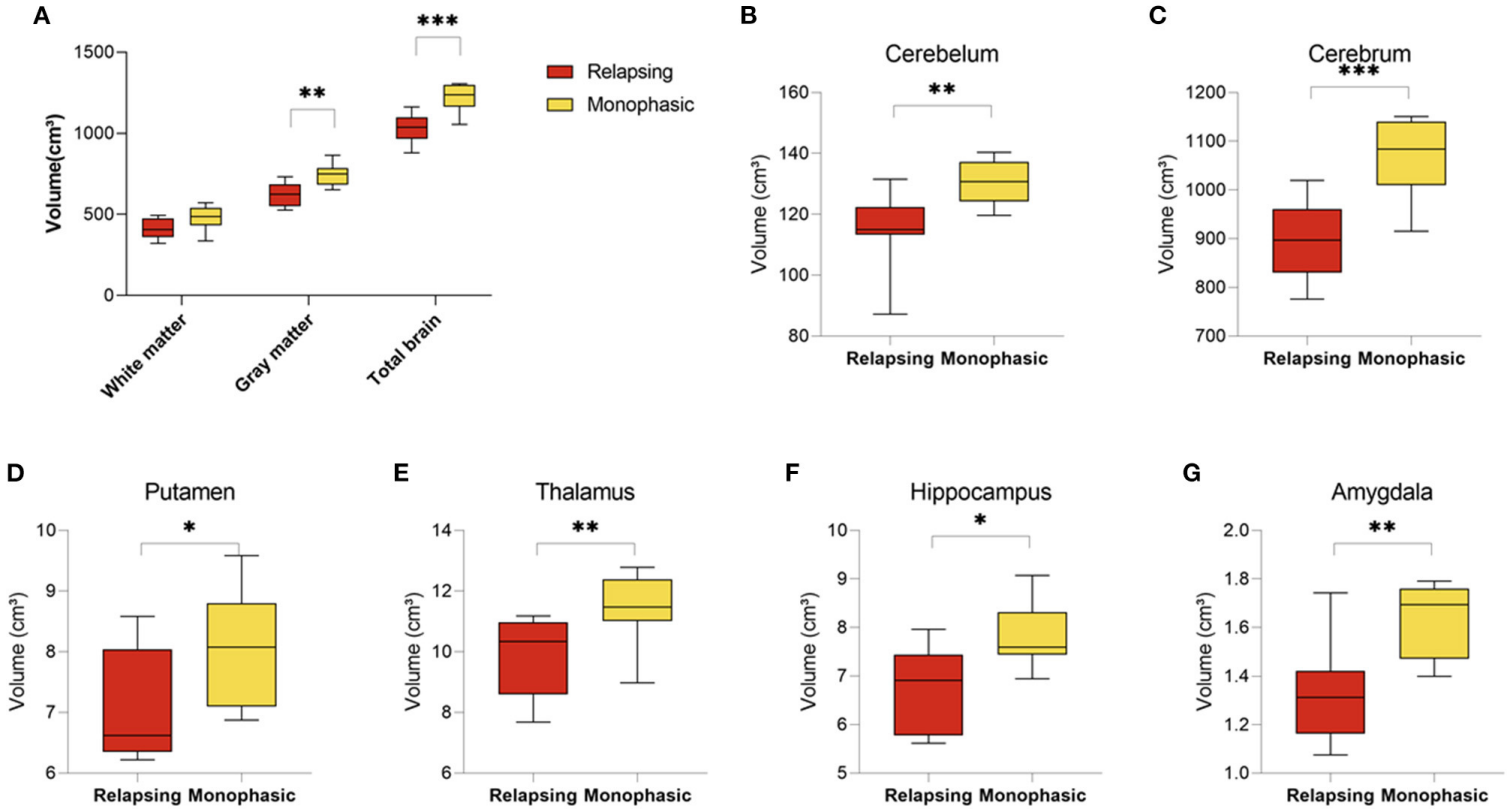
Decreased brain volume in relapsing compared to monophasic MOGAD patients. Brain volume of relapsing (*n* = 8) and monophasic MOGAD patients (*n* = 8) analyzed by Volbrain software. **(A)** White matter (413.63 ± 62.74 vs. 476.17 ± 74.35, *p* = 0.088), gray matter (620.94 ± 73.22 vs. 747.28 ± 68.61, *p* = 0.003) and whole brain volumes (1,034.07 ± 88.93 vs. 1,223.45 ± 86.84, *p* < 0.001), **(B)** cerebellum (114.95 ± 12.81 vs. 130.48 ± 7.08, *p* = 0.010), **(C)** cerebrum (899.04 ± 88.93 vs. 1,070.78 ± 86.84, *p* < 0.001), **(D)** Putamen (7.02 ± 0.94 vs. 8.04 ± 0.96, *p* = 0.051), **(E)** thalamus (9.88 ± 1.30 vs. 11.45 ± 1.19, *p* = 0.024), **(F)** hippocampus (6.72 ± 0.86 vs. 7.79 ± 0.68, *p* = 0.015), and **(G)** amygdala (1.33 ± 0.21 vs. 1.63 ± 0.15, *p* = 0.005) of monophasic and relapsing MOGAD patients. MOGAD, Myelin oligodendrocyte glycoprotein antibody disorders. *p* * ≤ 0.05, ** ≤ 0.01, *** ≤ 0.001.

MOGAD can follow a monophasic or relapsing course. Volumetric analysis of brain MRI scans revealed significant differences between patients presented with relapsing (*n* = 8) vs. monophasic disease course (*n* = 8). Patients with relapsing disease course presented with a significant decrease in brain volume (1,034.07 ± 88.93 vs. 1,223.45 ± 86.84, *p* < 0.001), gray matter volume (620.94 ± 73.22 vs. 747.28 ± 68.61, *p* = 0.003) and a trend toward a decreased white matter volume (413.63 ± 62.74 vs. 476.17 ± 74.35, *p* = 0.088) (Figure 3A). In addition, analysis of brain structures revealed significantly increased volume loss in the cerebellum (114.95 ± 12.81 vs. 130.48 ± 7.08, *p* = 0.010), cerebrum (899.04 ± 88.93 vs. 1,070.78 ± 86.84, *p* < 0.001), putamen (7.02 ± 0.94 vs. 8.04 ± 0.96, *p* = 0.051), thalamus (9.88 ± 1.30 vs. 11.45 ± 1.19, *p* = 0.024), hippocampus (6.72 ± 0.86 vs. 7.79 ± 0.68, *p* = 0.015), and amygdala (1.33 ± 0.21 vs. 1.63 ± 0.15, *p* = 0.005) in patients with relapsing disease course compared to monophasic disease course (Figures 3A–G) (Volumetric MRI parameters according to disease course are presented in Supplementary Table S4).

The differences between relapsing and monophasic patients were validated using the MDbrain program. MDbrain analysis calculates the coded deviations from normal database. In line with our findings in volBrain analysis, relapsing patients had decreased total brain volume (85.15 ± 14.49 vs. 30.79 ± 36.84, *p* = 0.021), white matter volume (91.90 ± 8.98 vs. 38.03 ± 39.19, *p* = 0.024), and hippocampus (70.00 ± 30.25 vs. 21.60 ± 15.83, *p* = 0.006) compared to monophasic patients (Supplementary Figures S1A–C).

**Figure 4:**
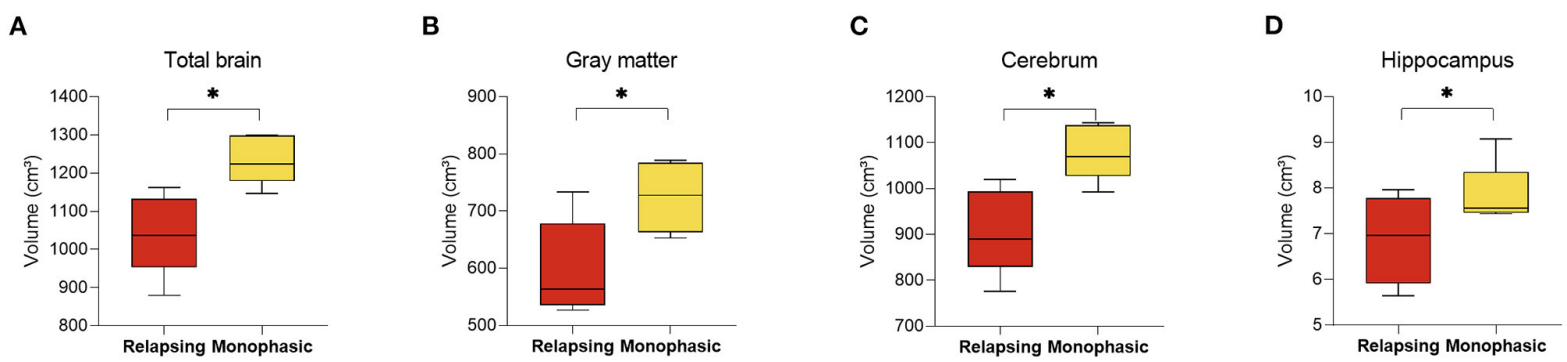
Decreased brain volume in relapsing compared to monophasic MOGAD patients during the first year of diagnosis. Brain volume of relapsing (*n* = 5) and monophasic MOGAD patients (*n* = 5) during the first year of diagnosis analyzed by Volbrain software. Volume of **(A)** total brain (1,235.95 ± 64.17 vs. 1,041.76 ± 105.77, *p* = 0.010), **(B)** gray matter volume (724.63 ± 61.14 vs. 598.34 ± 83.82, *p* = 0.038), **(C)** cerebrum (1,080.29 ± 61.15 vs. 907.26 ± 92.98, *p* = 0.010), and **(D)** hippocampus (7.83 ± 0.70 vs. 6.87 ± 0.96, *p* = 0.044) of monophasic and relapsing course MOGAD patients during the first year of diagnosis. MOGAD, Myelin oligodendrocyte glycoprotein antibody disorders. *p* * ≤ 0.05.

Analysis of MRIs performed during the first year of diagnosis revealed significant changes between MOGAD disease phenotypes (10 MRI scans were available from the first year following diagnosis: 5 monophasic and 5 relapsing disease course). Patients that will develop a relapsing MOGAD disease course had decreased whole brain (1,235.95 ± 64.17 vs. 1,041.76 ± 105.77, *p* = 0.010), along with gray matter volume (724.63 ± 61.14 vs. 598.34 ± 83.82, *p* = 0.038), cerebrum (1,080.29 ± 61.15 vs. 907.26 ± 92.98, *p* = 0.010), and hippocampus (7.83 ± 0.70 vs. 6.87 ± 0.96, *p* = 0.044) at diagnosis compared to patients who exhibit a monophasic disease course (Figures 4A–D). No significant changes were seen between the monophasic group and HCs.

Additional analysis revealed that patients who experienced more than one relapse during the first 3 years after diagnosis showed significantly decreased volume of total brain (1,025.55 ± 102.59 vs. 1,190.69 ± 103.66, *p* = 0.008), gray matter (602.60 ± 75.69 vs. 733.02 ± 68.23, *p* = 0.003), cerebrum (891.53 ± 91.65 vs. 1,040.94 ± 95.94, *p* = 0.008), cerebellum (113.92 ± 14.69 vs. 127.99 ± 8.45, *p* = 0.028), and thalamus (9.72 ± 1.50 vs. 11.23 ± 1.15, *p* = 0.040) compared to those who had only one relapse (Supplementary Figures S2A–E).

### Increased Deep Gray Matter Volume Loss in MOGAD Patients

#### Cerebellar Volume in MOGAD

Pursuant to our findings, we studied volume loss in the cerebellum and hippocampus in depth in MOGAD patients compared to HCs. The cerebellum is a major structure of the hindbrain that has an important role in motor control. The cerebellum lobules are divided into 3 functional divisions from motor (lobules I-VI, VIII) to attentional (VI, VIIB, IX) to default-mode processing (Crus I, Crus II and X).

**Figure 5:**
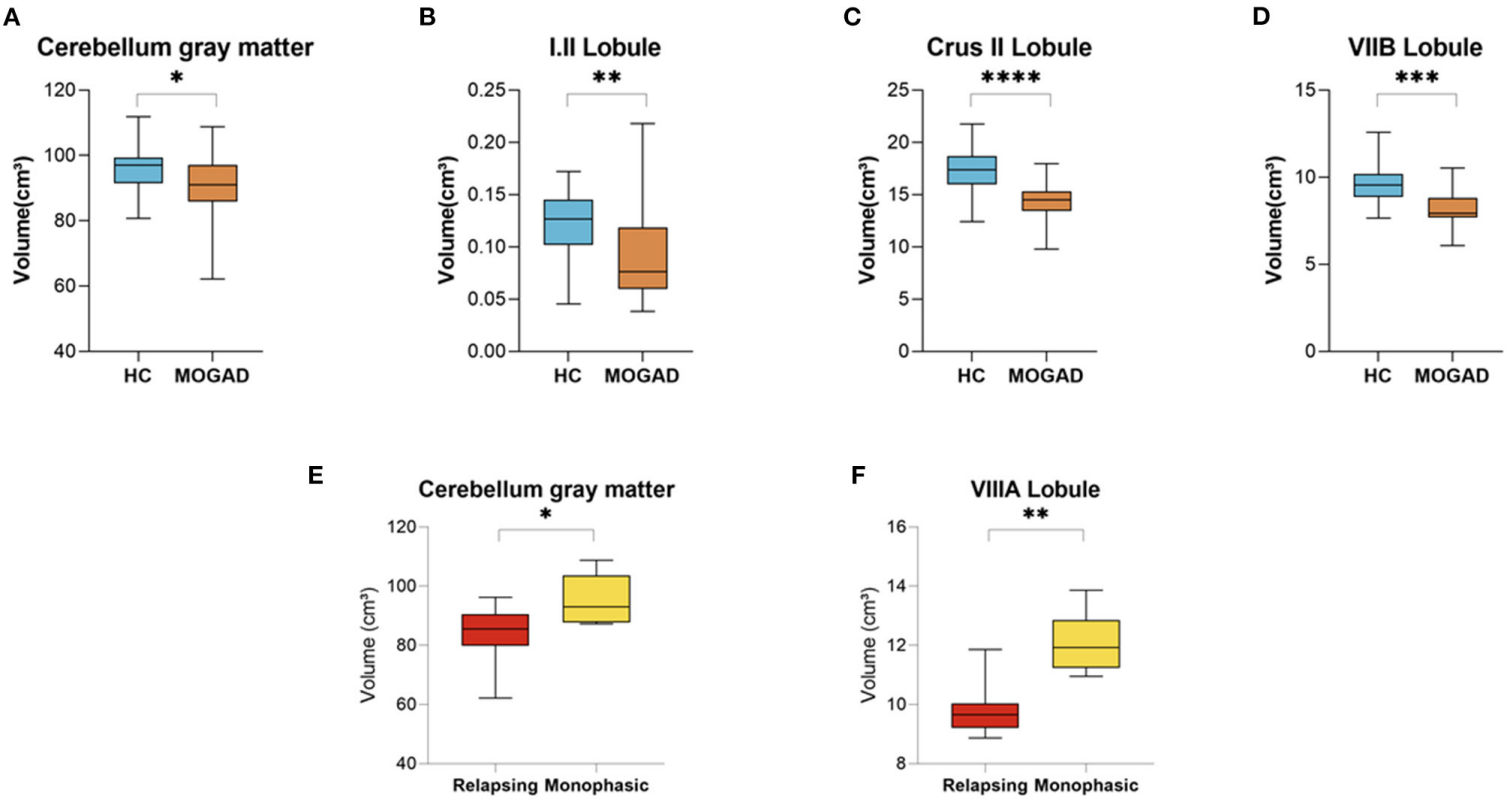
Decrease volume of cerebellar lobules at MOGAD patients compared to HCs and decreased volume of cerebellar lobules at relapsing MOGAD patients compared to monophasic MOGAD patients. **(A–D)** Volume of cerebellar lobules of HCs (*n* = 22) and MOGAD patients (*n* = 20) analyzed by CERES pipeline of Volbrain software. Volume of **(A)** total cerebellum gray matter (96.23 ± 6.89 vs. 90.66 ± 10.43, *p* = 0.050), **(B)** cerebellar lobules I.II (0.12 ± 0.03 vs. 0.09 ± 0.05, *p* = 0.009), **(C)** Crus II (17.33 ± 2.18 vs. 14.39 ± 1.75, *p* < 0.001), and **(D)** VIIB (9.70 ± 1.24 vs. 8.26 ± 1.09, *p* < 0.001) of MOGAD patients and HCs. **(E,F)** Volume of cerebellar lobules of monophasic (*n* = 6) and relapsing (*n* = 6) MOGAD patients analyzed by CERES pipeline of Volbrain software. Volume of **(E)** total cerebellum gray matter (83.14 ± 10.78 vs. 95.34 ± 8.52, *p* = 0.047) and **(F)** cerebellar lobule VIIIA (9.87 ± 0.97 vs. 12.09 ± 1.03, *p* = 0.002) of monophasic and relapsing MOGAD patients. MOGAD, Myelin oligodendrocyte glycoprotein antibody disorders; HCs, healthy controls. *p* * ≤ 0.05, ** ≤ 0.01, *** ≤ 0.001, **** ≤ 0.0001.

In our cohort of MOGAD patients we found a decreased volume of total cerebellum gray matter (96.23 ± 6.89 vs. 90.66 ± 10.43, *p* = 0.050) in addition to decreased volumes of the cerebellum lobules: I.II (0.12 ± 0.03 vs. 0.09 ± 0.05, *p* = 0.009), Crus II (17.33 ± 2.18 vs. 14.39 ± 1.75, *p* < 0.001), and VIIB (9.70 ± 1.24 vs. 8.26 ± 1.09, *p* < 0.001) compared to HCs (Figures 5A–D).

Relapsing MOGAD patients showed a significant decrease volume of total cerebellum gray matter (83.14 ± 10.78 vs. 95.34 ± 8.52, *p* = 0.047), and cerebellar lobule VIIIA (9.87 ± 0.97 vs. 12.09 ± 1.03, *p* = 0.002) compared to monophasic MOGAD patients (Figures 5E,F) (Volumetric cerebellar parameters are presented in Supplementary Tables S5,6).

#### Hippocampal Volume in MOGAD

The hippocampus is a complex, heterogeneous structure in the medial temporal lobe that plays an important role in the limbic system. Typically, it is divided into the subiculum, presubiculum, parasubiculum, the cornu ammonis (CA) fields 1–4 and the dentate gyrus (DG) (21). The CA can also be differently divided to 6 strata; stratum oriens (SO), stratum pyramidale (SP), stratum lucidum (SLU), stratum radiatum (SR), stratum lacunosum (SL) and the stratum molecuare (SM).

**Figure 6:**
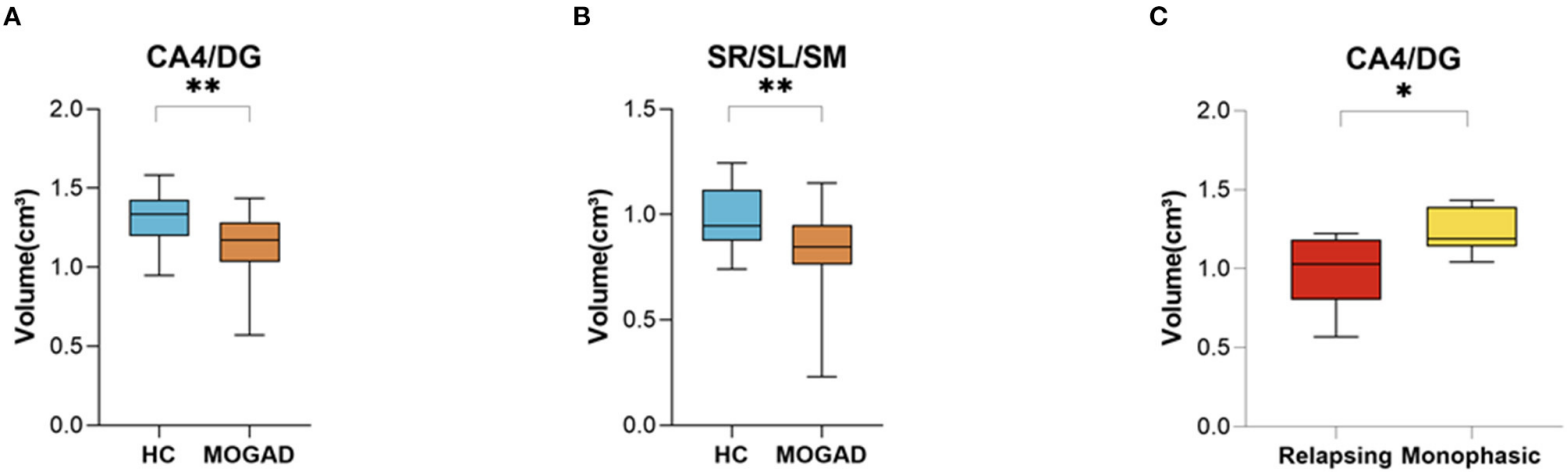
Decreased Volume of CA4/DG at MOGAD patients compare to HCs. **(A,B)** Volume of hippocampal subfields of HCs (*n* = 21) and MOGAD patients (*n* = 18) analyzed by HIPS pipeline of Volbrain software. **(A)** Volumes of CA/DG4 (1.31 ± 0.18 vs. 1.14 ± 0.21, *p* = 0.009) and **(B)** SR/SL/SM (0.98 ± 0.14 vs. 0.82 ± 0.22, *p* = 0.008). **(C)** Volume of hippocampal subfields of monophasic (*n* = 6) and relapsing patients (*n* = 6), analyzed by HIPS pipeline of Volbrain software. **(C)** CA4/DG (0.98 ± 0.24 vs. 1.23 ± 0.14, *p* = 0.03) volume of MOGAD monophasic and relapsing course patients. MOGAD, Myelin oligodendrocyte glycoprotein antibody disorders; HCs, healthy controls. *p* * ≤ 0.05, ** ≤ 0.01.

We assessed specific subregions of the hippocampus using the HIPS pipeline to determine the extent and pattern of hippocampal atrophy. Subregional analysis of MOGAD patients revealed increased volume loss in CA4/DG (1.31 ± 0.18 vs. 1.14 ± 0.21, *p* = 0.009) and in SR/SL/SM (0.98 ± 0.14 vs. 0.82 ± 0.22, *p* = 0.008) subfields in MOGAD patients compared to HCs (Figures 6A,B). In addition, patients with a relapsing disease course showed a significantly decreased volume in CA4/DG (0.98 ± 0.24 vs. 1.23 ± 0.14, *p* = 0.028) compared to patients with monophasic disease (Figure 6C) (Volumetric hippocampal parameters of MOGAD patients with different phenotypes are presented in Supplementary Tables S7,8).

#### Brain Atrophy in MOGAD Patients Correlates With Disease Severity

**Figure 7:**
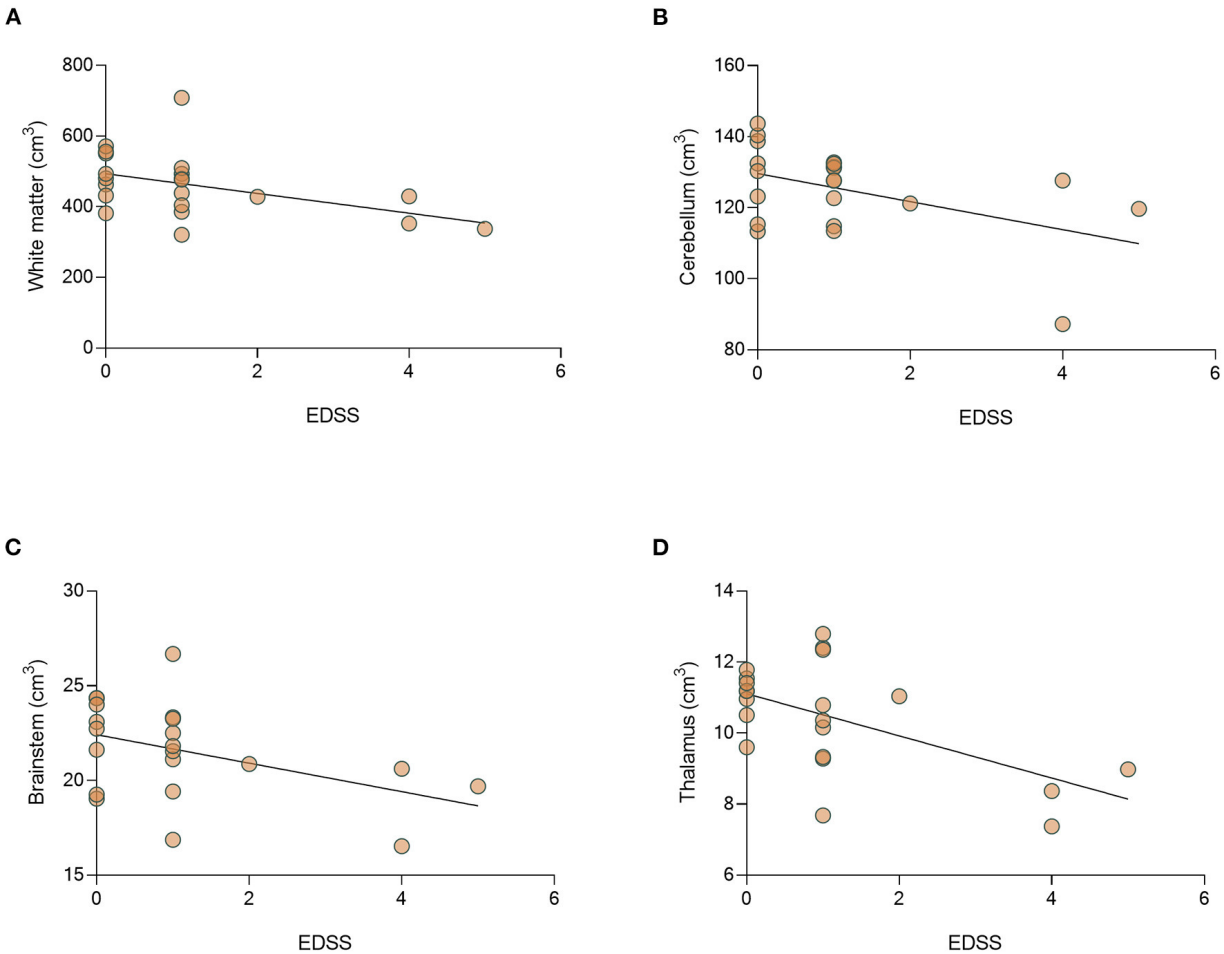
There is a significant correlation between EDSS and decrease volume of thalamus of MOGAD patients. Correlation between EDSS and brain volumes. Correlation between EDSS and volumes of **(A)** white matter (*r* = −0.051, *p* = 0.021), **(B)** cerebellum (*r* = −0.389, *p* = 0.082), **(C)** brainstem (*r* = −0.393, *p* = 0.078), and **(D)** thalamus (*r* = −0.476, *p* = 0.029) in MOGAD patients (*n* = 22). MOGAD, Myelin oligodendrocyte glycoprotein antibody disorders.

We then studied the correlation between disease severity (EDSS) and volumetric analysis. The median (range) EDSS in the cohort was 1 (0–5). Analysis of EDSS data showed a significant negative correlation between EDSS level and the volume of the white matter (*r* = −0.501, *p* = 0.021) and thalamus (*r* = −0.476, *p* = 0.029). In addition, there is a trend toward a negative correlation between EDSS and the volume of cerebellum (*r* = −0.389, *p* = 0.082), and brainstem (*r* = −0.393, *p* = 0.078) (Figures 7A–D). No differences in brain volume were found between patients with ON or those with myelitis. In addition, we found that relapse number, but not disease duration at the time of MRI performing significantly correlated to decreased total brain volume (*r* = −0.573, *p* = 0.007, Supplementary Figure S3).

## Discussion

Volumetric analysis of brain MRI scans revealed significantly increased brain volume loss in MOGAD patients compared to HCs, with significantly decreased volume of deep gray matter structures: cerebellum, brainstem, caudate, thalamus, hippocampus and amygdala. Moreover, we found a strong association between brain volume loss and disease phenotype. In our cohort of MOGAD, patients with relapsing disease course presented with increased total brain volume loss early after diagnosis, with specifically increased atrophy of the cerebellum and hippocampus compared to patients with monophasic disease course.

According to recent publications, MOGAD patients tend to present with lesions in the cortical and subcortical deep gray matter, white matter, brainstem, cerebellum, and spinal cord (13). MOGAD pathology is dominated by coexistence of both perivenous and confluent white matter demyelination, with an overrepresentation of intracortical demyelinated lesions compared to typical MS (10). To date, there are few works studying brain volume of MOGAD patients (22–24). Zhuo et al. described gray matter atrophy in both the frontal and temporal lobes, insula, thalamus, and hippocampus, and white matter fiber disruption in the optic radiation and anterior/posterior corona radiate (22). Messina et al. showed decreased deep gray matter volume in MS and MOGAD patients, compared to HCs (24). In contrast, Schmidt et al. did not identify significant differences in brain volume between MOGAD patients and HCs (23). In accordance with the former groups, in our cohort of MOGAD patients, we found decreased total brain, white matter, cerebellum, brainstem, caudate, thalamus, hippocampus and amygdala volume compared to HCs. Increased brain volume loss is known to occur in other demyelinating diseases, including in both MS and NMOSD, with increased severity in MS (25).

Deep gray matter atrophy was observed in all MOGAD patients in our cohort, with significant increases in patients with a relapsing phenotype. In MS patients, the involvement of the gray matter, particularly of the thalamus, has been linked to a wide range of clinical manifestations including cognitive decline, motor deficits, fatigue, painful syndromes, and ocular motility disturbances (26). Gray matter atrophy has been identified, particularly in patients with secondary-progressive MS compared to relapsing-remitting MS, is associated with T2 and T1 lesion volume, and correlates with physical and cognitive impairment (27). The volumes of the deep gray matter were reduced in MS compared to NMOSD (28), and in NMOSD deep gray matter atrophy is restricted to the thalamus (although broadly distributed in MS). In the current study we found that, as in MS and NMOSD, there is significant thalamic atrophy in MOGAD patients. In addition, the thalamic atrophy correlated with EDSS, as described in MS and NMOSD (29, 30). Suggesting that gray matter atrophy could be a possible biomarker for disease severity in inflammatory demyelinating diseases.

To date, we still do not have a reliable biomarker to predict disease course of MOGAD. About 40–55% of patients will follow a monophasic course and might not need preventive therapy (31). In this study we found significant volumetric changes between disease phenotypes, early during the first year after diagnosis. Relapsing patients presented with significantly decreased total brain volume and deep gray matter (cerebrum, putamen, thalamus, hippocampus, and amygdala) in the first year compared to monophasic MOGAD patients. Identifying disease course early after diagnosis will allow for optimized treatment. Suggesting a potential use of MRI volumetry as a biomarker for predicting a relapse course and/or a short interval between the first and second relapse in patients with MOGAD.

In our cohort we found that there is a negative correlation between number of relapses and brain volume, without correlation to disease duration. A recent study shows that new remission silent lesions are found only in 3% of MOGAD patients (27). It is therefore possible that brain atrophy in MOGAD patients might be a result of multiple relapses rather than disease progression over time.

Due to its multiple connections to the forebrain, the thalamus and the spinal cord, the cerebellum is not only affected by focal white and gray matter lesions but also by the secondary degeneration of multiple afferent and efferent connections to the supratentorial brain areas and to the spinal cord. Hence, cerebellar atrophy might occur at a significant rate with higher chances of affecting the patient's clinical outcome due to the cerebellar strategic position in the motor, coordination, and cognitive networks (32). Damage to the cerebellum is associated with dysdiadokokinesia, ataxia, tremors, loss of balance, muscle weakness, dysarthria, and loss of postural tone. There is also evidence supporting cognitive function of the cerebellum (32).

In MS, tissue damage within the cerebellum is thought to contribute to disability (33). Cerebellar atrophy has been found to be more extensive in patients with secondary progressive MS and correlates with disease duration and disability (33). Hippocampal atrophy also begins early in MS, as shown in MRI studies, and this atrophy has been correlated with impaired performance on visuospatial memory testing, commonly affected in MS patients (34). In NMO, the MRI predictor of cognitive functions is the hippocampal volume (35).

The hippocampus is located in the medial temporal lobe at both sides of the brainstem near to the cerebellum, and is critical for memory functions (21). CA4/DG subfield is the first subfield to be atrophied across the course of MS, at the stage of clinical isolate syndrome; atrophy then spreads to CA1 (34). Furthermore, the CA/DG subfield is significantly decreased in NMO and anti-N-methyl-D-aspartate receptor encephalitis patients compared to HCs (36, 37). Interestingly, in our cohort of total MOGAD patients, and specifically in relapsing patients, the most affected subfield of the hippocampus is the CA4/DG subfield.

Limitations of this study include the small sample size due to the low incidence of the disease, and short follow-up time. Despite these limitations, our findings show for the first-time volumetric brain differences between the monophasic and relapsing MOGAD patients.

In conclusion, in the current study, we found volumetric differences between MOGAD patients that present with relapsing and monophasic disease course. In addition, we identified correlations between disease severity and thalamus atrophy. As early as the first year after diagnosis, patients with relapsing disease course have significant decreased total brain and lower cerebrum and hippocampus volume compared to patients with monophasic disease course. Differences between MOGAD patients and HCs were found, especially at the deep gray matter structures.

## Data Availability Statement

The raw data supporting the conclusions of this article will be made available by the authors, without undue reservation.

## Ethics Statement

The studies involving human participants were reviewed and approved by Hadassah Medical Organization's Ethics Committee (reference no. HMO-20-0644). Written informed consent from the participants was not required to participate in this study in accordance with the national legislation and the institutional requirements.

## Author Contributions

AR contributed to study design, clinical and MRI data acquisition, data analysis and interpretation, and manuscript preparation. LB and AV-D contributed to study design, data analysis and interpretation, and manuscript preparation. OZ and NH contributed to data acquisition. BU contributed to clinical data acquisition and patient recruitment. AB and NL contributed to MRI data acquisition. All authors read and approved the final manuscript.

## Conflict of Interest

The authors declare that the research was conducted in the absence of any commercial or financial relationships that could be construed as a potential conflict of interest.

## Publisher's Note

All claims expressed in this article are solely those of the authors and do not necessarily represent those of their affiliated organizations, or those of the publisher, the editors and the reviewers. Any product that may be evaluated in this article, or claim that may be made by its manufacturer, is not guaranteed or endorsed by the publisher.
