# Supplementary material for: Volumetric Brain Loss Correlates With a Relapsing MOGAD Disease Course
Source: Front Neurol. 2022 Mar 24;13:867190. doi: 10.3389/fneur.2022.867190 (PMC8987978; doi:10.3389/fneur.2022.867190)
Supplement: Supplementary file 1 [file Table_1.DOCX]

Supplementary Table 1: Volumetric brain MRI parameters of MOGAD patients performed with 1.5 and 3 tesla MRI scanner.

| Variable volume, cm³ | brain MRI performed with 1.5 Tesla MRI scanner (n=9) mean±SD | brain MRI performed with 3 Tesla MRI scanner (n=13) mean±SD | P value |
| --- | --- | --- | --- |
| Total Brain | 1127.75±136.61 | 1148.33±142.45 | 0.738 |
| Gray matter | 659.00±100.59 | 696.61±112.04 | 0.430 |
| White matter | 468.75±124.00 | 451.71±60.61 | 0.672 |
| CSF | 172.68±72.49 | 186.18±84.19 | 0.852 |
| Cerebrum | 982.38±119.86 | 1002.32±134.11 | 0.724 |
| Cerebellum | 124.15±16.00 | 124.18±9.97 | 0.996 |
| Brainstem | 21.26±3.51 | 21.86±1.45 | 0.584 |
| Lateral ventricles | 14.64±7.07 | 12.43±11.17 | 0.607 |
| Caudate | 6.18±0.78 | 6.87±0.89 | 0.091 |
| Putamen | 7.34±1.04 | 8.21±1.97 | 0.240 |
| Thalamus | 10.11±1.38 | 11.17±1.86 | 0.162 |
| Globus pallidus | 2.09±0.22 | 2.26±0.57 | 0.414 |
| Hippocampus | 6.99±1.12 | 6.76±1.40 | 0.689 |
| Amygdala | 1.42±0.17 | 1.33±0.45 | 0.566 |
| Nucleus accumbens | 0.63±0.15 | 0.68±0.28 | 0.625 |

Independent t Test was used to compare the means of the two groups. P < 0.05 was considered as significant.

MOGAD: Myelin oligodendrocyte glycoprotein antibody disorders
