# Supplementary material for: Volumetric Brain Loss Correlates With a Relapsing MOGAD Disease Course
Source: Front Neurol. 2022 Mar 24;13:867190. doi: 10.3389/fneur.2022.867190 (PMC8987978; doi:10.3389/fneur.2022.867190)
Supplement: Supplementary file 2 [file Table_2.DOCX]

Supplementary Table 3: Volumetric brain MRI parameters of MOG-AD patients and HCs

| Variable volume, cm³ | HCs (n=22) mean±SD | MOG (n=22) mean±SD | P Value |
| --- | --- | --- | --- |
| Total Brain | 1214.33±103.11 | 1139.91±137.16 | **0.048** |
| Gray matter | 699.97±59.45 | 681.66±106.70 | 0.476 |
| White matter | 514.36±56.59 | 458.68±89.60 | **0.018** |
| CSF | 178.89±48.90 | 180.66±78.09 | 0.929 |
| Cerebrum | 1056.36±95.23 | 994.16±125.90 | 0.072 |
| Cerebellum | 134.03±10.44 | 124.17±12.42 | **0.007** |
| Brainstem | 23.96±2.49 | 21.61±2.45 | **0.003** |
| Lateral ventricles | 12.42±12.68 | 13.33±9.57 | 0.788 |
| Caudate | 7.49±0.78 | 6.56±1.03 | **0.001** |
| Putamen | 8.65±1.05 | 7.85±1.68 | 0.067 |
| Thalamus | 11.92±0.99 | 10.74±1.72 | **0.008** |
| Globus pallidus | 2.37±0.28 | 2.19±0.46 | 0.125 |
| Hippocampus | 7.72±0.85 | 6.86±1.27 | **0.011** |
| Amygdala | 1.60±0.27 | 1.37±0.36 | **0.024** |
| Nucleus accumbens | 0.71±0.12 | 0.66±0.23 | 0.329 |

Independent t Test was used to compare the means of the two groups. P < 0.05 was considered as significant.

MOGAD: Myelin oligodendrocyte glycoprotein antibody disorders; HCs: healthy controls
