# Supplementary material for: Volumetric Brain Loss Correlates With a Relapsing MOGAD Disease Course
Source: Front Neurol. 2022 Mar 24;13:867190. doi: 10.3389/fneur.2022.867190 (PMC8987978; doi:10.3389/fneur.2022.867190)
Supplement: Supplementary file 3 [file Table_3.DOCX]

Supplementary Table 4: Volumetric brain MRI parameters of MOG-AD patients during first year after onset and HCs

| Variable volume, cm³ | HCs (n=22) mean±SD | MOGAD (n=15) mean±SD | P Value |
| --- | --- | --- | --- |
| Total Brain | 1214.33±103.11 | 1169.15±122.74 | 0.234 |
| Gray matter | 699.97±59.45 | 677.02±106.45 | 0.407 |
| White matter | 514.36±56.59 | 492.12±83.63 | 0.340 |
| CSF | 178.89±48.90 | 170.62±58.96 | 0.645 |
| Cerebrum | 1056.36±95.23 | 1020.86±110.50 | 0.304 |
| Cerebellum | 134.03±10.44 | 126.18±13.84 | 0.057 |
| Brainstem | 23.96±2.49 | 22.13±2.48 | **0.035** |
| Lateral ventricles | 12.42±12.68 | 11.72±5.36 | 0.841 |
| Caudate | 7.49±0.78 | 6.68±1.03 | **0.010** |
| Putamen | 8.65±1.05 | 8.21± 1.88 | 0.369 |
| Thalamus | 11.92±0.99 | 10.99±1.67 | **0.040** |
| Globus pallidus | 2.37±0.28 | 2.19±0.48 | 0.158 |
| Hippocampus | 7.72±0.85 | 6.82±1.41 | **0.021** |
| Amygdala | 1.60±0.27 | 1.32±0.41 | **0.021** |
| Nucleus accumbens | 0.71±0.12 | 0.63±0.17 | 0.173 |

Independent t Test was used to compare the means of the two groups. P < 0.05 was considered as significant.

MOGAD: Myelin oligodendrocyte glycoprotein antibody disorders; HCs: healthy controls
