# Supplementary material for: Volumetric Brain Loss Correlates With a Relapsing MOGAD Disease Course
Source: Front Neurol. 2022 Mar 24;13:867190. doi: 10.3389/fneur.2022.867190 (PMC8987978; doi:10.3389/fneur.2022.867190)
Supplement: Supplementary file 4 [file Table_4.DOCX]

Supplementary Table 5: Volumetrics brain MRI parameters of MOGAD relapsing and monophasic disease course

| Variable volume, cm³ | Relapsing (n=8) mean±SD | Mono (n=8) mean±SD | P value |
| --- | --- | --- | --- |
| Total Brain | 1034.07±88.93 | 1223.45±86.84 | **>0.001** |
| Gray matter | 620.94±73.22 | 747.28±68.61 | **0.003** |
| White matter | 413.13±62.74 | 476.17±74.35 | 0.088 |
| CSF | 206.55±64.47 | 187.55±91.66 | 0.644 |
| Cerebrum | 899.04±79.19 | 1070.78±81.65 | **<0.001** |
| Cerebellum | 114.95±12.81 | 130.48±7.08 | **0.010** |
| Brainstem | 20.12±2.41 | 22.25±1.89 | 0.070 |
| Lateral ventricles | 12.55±7.64 | 18.48±12.44 | 0.270 |
| Caudate | 6.13±0.73 | 6.78±0.99 | 0.156 |
| Putamen | 7.02±0.94 | 8.04±0.96 | 0.051 |
| Thalamus | 9.88±1.30 | 11.45±1.19 | **0.024** |
| Globus pallidus | 2.11±0.37 | 2.45±0.48 | 0.131 |
| Hippocampus | 6.72±0.86 | 7.79±0.68 | **0.015** |
| Amygdala | 1.33±0.21 | 1.63±0.15 | **0.005** |
| Nucleus accumbens | 0.62±0.12 | 0.75±0.25 | 0.215 |

Independent t Test was used to compare the means of the two groups. P < 0.05 was considered as significant.

MOGAD: Myelin oligodendrocyte glycoprotein antibody disorders
