# Supplementary material for: Volumetric Brain Loss Correlates With a Relapsing MOGAD Disease Course
Source: Front Neurol. 2022 Mar 24;13:867190. doi: 10.3389/fneur.2022.867190 (PMC8987978; doi:10.3389/fneur.2022.867190)
Supplement: Supplementary file 5 [file Table_5.DOCX]

Supplementary Table 6: Volumetric cerebellar MRI parameters of MOG-AD patients and HCs

| Variable volume, cm³ | HC (n=22) mean±SD | MOG (n=20) mean±SD | P value |
| --- | --- | --- | --- |
| Cerebellum cortical thickness | 4.64±0.37 | 4.76±0.21 | 0.206 |
| Cerebellum gray matter | 96.23±6.89 | 90.66±10.43 | **0.050** |
| I.II cerebellar lobule | 0.12±0.03 | 0.09±0.05 | **0.009** |
| III cerebellar lobule | 1.38±0.20 | 1.52±0.33 | 0.113 |
| IV cerebellar lobule | 4.26±0.70 | 4.29±0.62 | 0.891 |
| V cerebellar lobule | 7.75±1.11 | 7.81±0.94 | 0.851 |
| VI cerebellar lobule | 17.52±1.80 | 16.81±2.08 | 0.261 |
| Crus I cerebellar lobule | 25.59±2.67 | 25.47±3.46 | 0.900 |
| Crus II cerebellar lobule | 17.33±2.18 | 14.39±1.75 | **0.001>** |
| VIIB cerebellar lobule | 9.70±1.24 | 8.26±1.09 | **0.001>** |
| VIIIA cerebellar lobule | 12.02±1.70 | 11.15±1.42 | 0.092 |
| VIIIB cerebellar lobule | 7.73±1.05 | 7.91±1.25 | 0.626 |
| IX cerebellar lobule | 7.29±0.92 | 7.07±1.13 | 0.500 |
| X cerebellar lobule | 1.29±0.18 | 1.24±0.20 | 0.383 |

Independent t Test was used to compare the means of the two groups. P < 0.05 was considered as significant.

MOGAD: Myelin oligodendrocyte glycoprotein antibody disorders; HCs: healthy controls
