# Supplementary material for: Volumetric Brain Loss Correlates With a Relapsing MOGAD Disease Course
Source: Front Neurol. 2022 Mar 24;13:867190. doi: 10.3389/fneur.2022.867190 (PMC8987978; doi:10.3389/fneur.2022.867190)
Supplement: Supplementary file 6 [file Table_6.DOCX]

Supplementary Table 7: Volumetric cerebellar MRI parameters of MOG-AD relapsing and monophasic disease course

| Variable volume, cm³ | Relapsing (n=6) mean±SD | Monophasic (n=6) mean±SD | P value |
| --- | --- | --- | --- |
| Cerebellum cortical thickness | 4.69±0.23 | 4.79±0.27 | 0.479 |
| Cerebellum gray matter | 83.14±10.78 | 95.34±8.52 | 0.047 |
| I.II cerebellar lobule | 0.10±0.06 | 0.09±0.04 | 0.686 |
| III cerebellar lobule | 1.37±0.29 | 1.49±0.29 | 0.450 |
| IV cerebellar lobule | 3.97±0.70 | 4.37±0.47 | 0.257 |
| V cerebellar lobule | 7.24±1.12 | 8.13±0.44 | 0.095 |
| VI cerebellar lobule | 15.58±2.66 | 17.69±1.41 | **0.110** |
| Crus I cerebellar lobule | 23.29±3.97 | 26.66±2.75 | 0.109 |
| Crus II cerebellar lobule | 13.67±1.93 | 14.74±2.06 | 0.355 |
| VIIB cerebellar lobule | 7.72±0.92 | 8.95±1.21 | 0.061 |
| VIIIA cerebellar lobule | 9.87±0.97 | 12.09±1.03 | **0.002** |
| VIIIB cerebellar lobule | 7.17±1.28 | 8.38±0.88 | 0.076 |
| IX cerebellar lobule | 6.58±0.69 | 7.22±0.82 | 0.151 |
| X cerebellar lobule | 1.15±0.21 | 1.30±0.16 | **0.195** |

Independent t Test was used to compare the means of the two groups. P < 0.05 was considered as significant.

MOGAD: Myelin oligodendrocyte glycoprotein antibody disorders
