# Supplementary material for: Volumetric Brain Loss Correlates With a Relapsing MOGAD Disease Course
Source: Front Neurol. 2022 Mar 24;13:867190. doi: 10.3389/fneur.2022.867190 (PMC8987978; doi:10.3389/fneur.2022.867190)
Supplement: Supplementary file 7 [file Table_7.DOCX]

Supplementary Table 8: Hippocampal volumes of MOG-AD patients and HCs

| Variable volume, cm³ | HC (n=21) mean±SD | MOG (n=18) mean±SD | P value |
| --- | --- | --- | --- |
| CA1 Subfield | 1.74±0.24 | 1.67±0.57 | 0.578 |
| CA2/CA3 Subfield | 0.34±0.24 | 0.30±0.09 | 0.106 |
| CA4/DG Subfield | 1.31±0.18 | 1.14±0.21 | **0.009** |
| SR/SL/SM Subfield | 0.98±0.14 | 0.82±0.22 | **0.008** |
| Subiculum | 0.58±0.08 | 0.55±0.10 | 0.372 |

Independent t Test was used to compare the means of the two groups. P < 0.05 was considered as significant.

MOGAD: Myelin oligodendrocyte glycoprotein antibody disorders; HCs: healthy controls
