# Supplementary material for: Volumetric Brain Loss Correlates With a Relapsing MOGAD Disease Course
Source: Front Neurol. 2022 Mar 24;13:867190. doi: 10.3389/fneur.2022.867190 (PMC8987978; doi:10.3389/fneur.2022.867190)
Supplement: Supplementary file 8 [file Table_8.DOCX]

Supplementary Table 9: Volumetrics hippocampal MRI parameters of MOG-AD relapsing and monophasic disease course

| Variable volume, cm³ | Relapsing (n=6) mean±SD | Monophasic (n=6) mean±SD | P value |
| --- | --- | --- | --- |
| CA1 Subfield | 1.40±0.36 | 1.61±0.34 | 0.305 |
| CA2/CA3 Subfield | 0.30±0.07 | 0.37±0.05 | 0.067 |
| CA4/DG Subfield | 0.98±0.24 | 1.23±0.14 | **0.028** |
| SR/SL/SM Subfield | 0.74±0.27 | 0.83 ±0.20 | 0.441 |
| Subiculum | 0.51±0.10 | 0.58±0.06 | 0.171 |

Independent t Test was used to compare the means of the two groups. P < 0.05 was considered as significant.

MOGAD: Myelin oligodendrocyte glycoprotein antibody disorders
