# Supplementary figures and images for: Volumetric Brain Loss Correlates With a Relapsing MOGAD Disease Course
Source: Front Neurol. 2022 Mar 24;13:867190. doi: 10.3389/fneur.2022.867190 (PMC8987978; doi:10.3389/fneur.2022.867190)

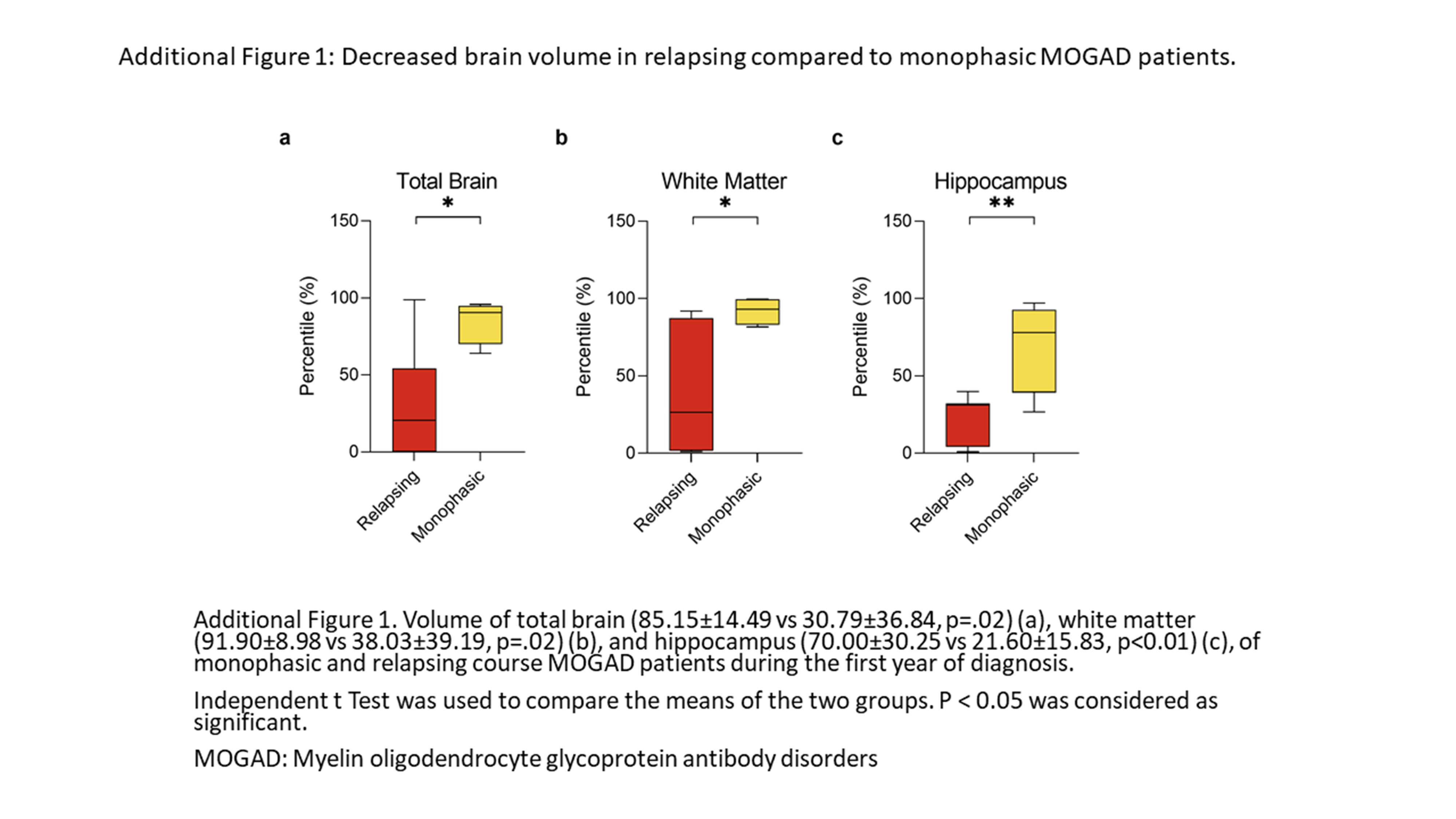

Supplement: Supplementary file 9 [file Image_1.TIF]

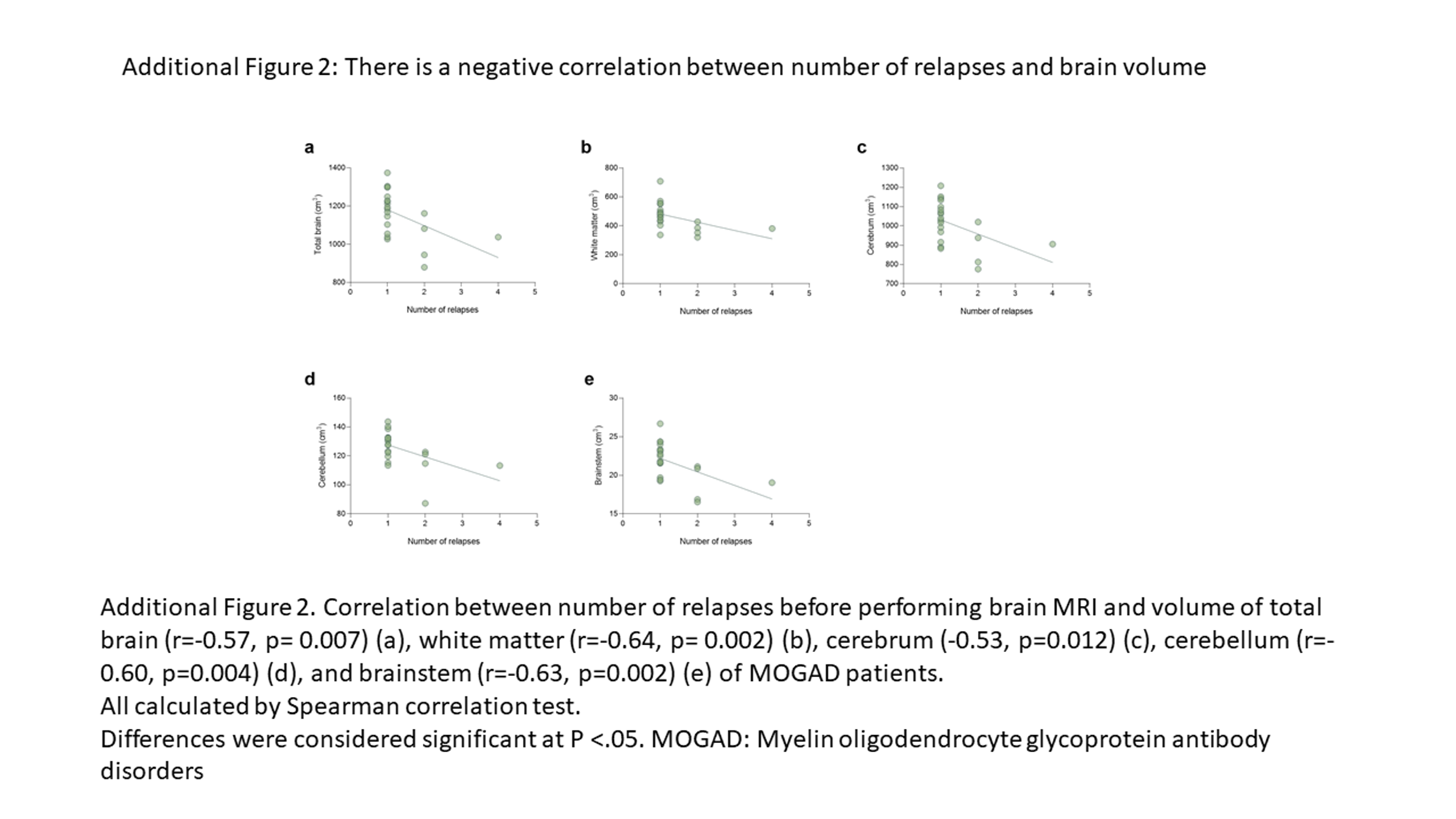

Supplement: Supplementary file 10 [file Image_2.TIF]

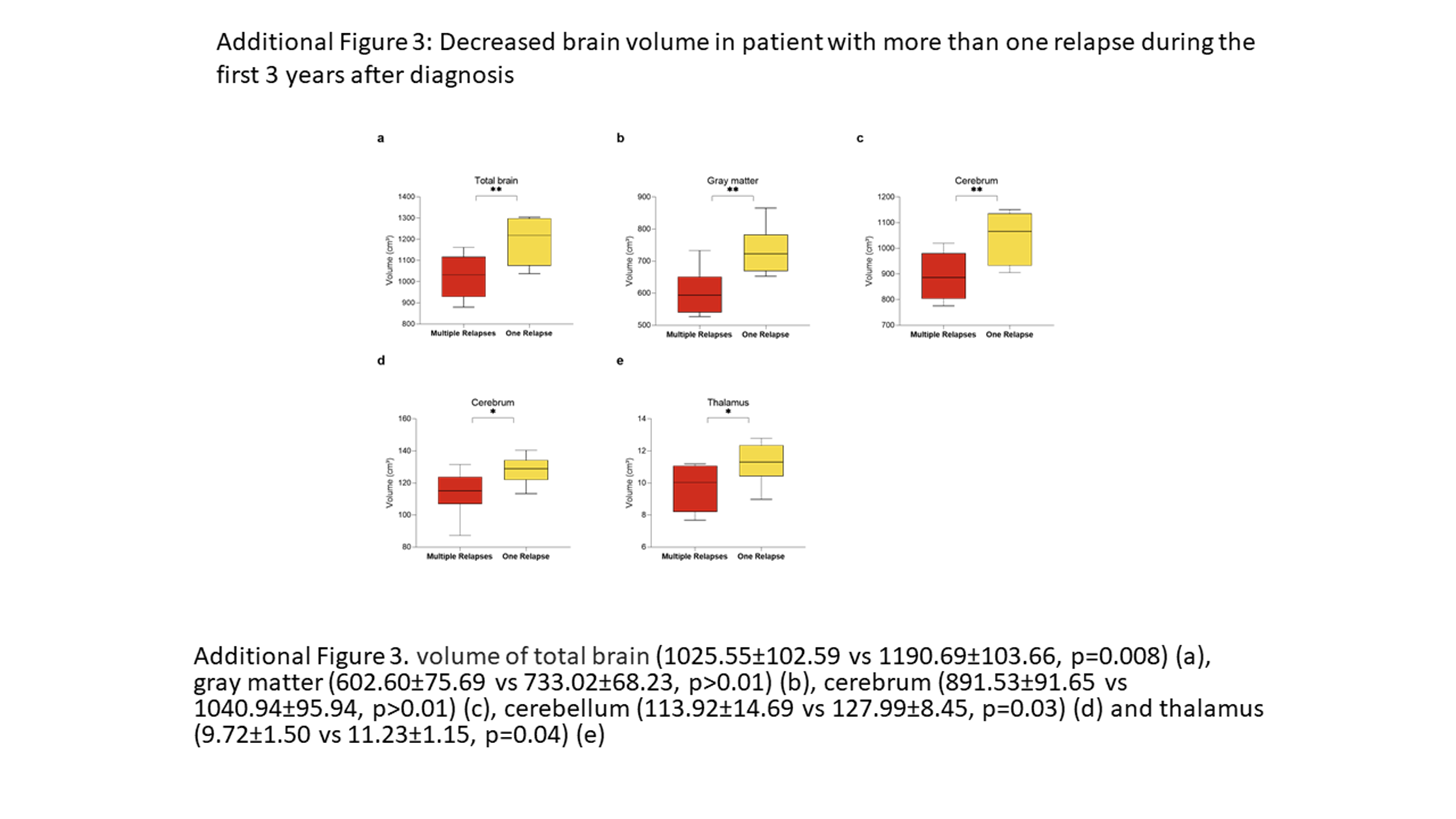

Supplement: Supplementary file 11 [file Image_3.TIF]
